# Improved Antioxidant Capacity of Optimization of a Self-Microemulsifying Drug Delivery System for Resveratrol

**DOI:** 10.3390/molecules201219750

**Published:** 2015-11-27

**Authors:** Ying Chen, Huiyong Zhang, Jing Yang, Haiyan Sun

**Affiliations:** 1 College of Life Sciences, Henan Normal University, Xinxiang 453007, China; 2 College of Life Science and Biotechnology, Xinxiang Medical University, Xinxiang 453003, China; huiyongzhang115@163.com; 3 School of Life Science and Technology, Henan Institute of Science and Technology, Xinxiang 453003, China; yangjing_228@sohu.com (J.Y.); haiyan20050101@163.com (H.S.)

**Keywords:** resveratrol, nano-encapsulation, self-emulsifying, oil-in-water, antioxidant, cytotoxicity

## Abstract

The use of nano-encapsulated resveratrol (RSV) in self-micro-emulsified drug delivery systems (SMEDDS) formulations was investigated. Self-emulsifying grading tests were used to establish the optimal ratio of oil, surfactant, and co-surfactant. The optimized system was further investigated for the droplet size and zeta potential at the different medium pH values by a Malvern Zetasizer and transmission electron microscopy (TEM). The antioxidant capacity and cytotoxicity of the formulation were detected by DCFH-DA and a CCK-8 assays. The results showed that the nano-emulsion based on ethyl oleate, Tween-80, and PEG-400 (35:40:25, *w*/*w*/*w*) was the most stable formulation due to the small droplet size (approximately 50 nm) and high zeta potential in a neutral environment. Furthermore, this formulation also exhibited a greater antioxidant capacity with less toxicity than free RSV. Taken together, considering these results and the simple fabrication process, this formulation could be used to deliver nutritional food supplements in a stable, efficient, and safe manner.

## 1. Introduction

The “French paradox”, namely consuming more saturated fat but with a relatively low incidence of cardiovascular diseases, is associated with the daily consumption of plant-derived polyphenolic phytochemicals found in red wine, such as resveratrol (3,4′,5-trihydroxystilbene, RSV) [1], which has two isomers, *i.e.*, *trans*-RSV and *cis*-RSV. As a result, RSV has been intensively investigated for its health-beneficial effects, including those combating inflammation, oxidative stress, cancer, and neurodegenerative diseases [2,3,4]. Based on these aforementioned beneficial effects, RSV has long been used either as a nutritional supplements or considered a medicinal candidate. However, contrary to the relatively high solubility of RSV in red wine (0.19 mg/mL), RSV exhibits extremely poor aqueous solubility (0.03 mg/mL in H_2_O at 37 °C). Furthermore, as a highly hydrophobic molecule, RSV also shows a high oil-water partition coefficient (log P_o/w_ 3.1) and a low absorption ratio (approximately 20%) [5]. As a result, these features limit RSV’s bioavailability at a low bio-efficacious concentration irrespective of the RSV formulation forms used, such as tablets, capsules, and powders [6].

To overcome these limitations, many strategies have been used to improve the bio-availability of poorly aqueous soluble compounds. In recent years, several RSV encapsulations have been produced, including liposomes, solid lipid nanoparticles, polymeric nanoparticles, and cyclodextrins, which were superior to free RSV and improved the aqueous solubility, bioavailability, stability, retention time, and tissue targeting [7,8]. In addition to these encapsulated formulations, self-micro-emulsified drug delivery systems (SMEDDS), which contained an isotropic mixture of oil, surfactant, and co-surfactant, have recently emerged as a promising approach due to their spontaneous formation of a stable oil-in-water emulsion state both *in vitro* and *in vivo* [9,10,11,12]. Besides, SMEDDS are suitable carriers for poorly water-soluble lipophilic agents, conferring protection to the incorporated agents, which are solubilized either in the hydrophobic core (the oil phase) or the hydrophilic shell (the hydrophilic head group layer of surfactant).

In fact, several researchers have shown that SMEDDS can improve RSV’s solubility and transport across the plasma membrane to enhance its effects within cells during the digestive process [9,13,14]. In particular, the biggest advantage of this formulation is that the microemulsions were isotropic and thermodynamically stable in the gastrointestinal tract (GI) in a dissolved state [15,16]. When the oil phase comes into the aqueous environment in the GI tract, it must emulsify immediately to form small particles, which resulted in better encapsulated compound release by providing a large interfacial area through which the compound could diffuse into the gastrointestinal fluid and thus increase its absorption. For this reason, it is urgent to develop an efficient SMEDDS to protect RSV and to enhance its bioavailability after oral administration. The droplet size is the main property in determining the release and absorption of hydrophobic compounds. It has been demonstrated that the particles which are less than 200 nm in size with hydrophilic surfaces, exhibit an improved enhanced permeability and retention (EPR) effect. Accordingly, in previous studies, the diameter of microencapsulated RSV was developed about 200 nm in SMEDDS formulations [9]. The main goal of this study was to develop an optimized RSV SMEDDS at a nano/micrometre level (<100 nm) with less cytotoxity to enable further use of RSV in medicines, supplements, and nutraceuticals. To evaluate the quality of the RSV nanoparticles, the optimal formulation was characterized by assessing the droplet size, morphology, polydispersity index (PDI), zeta potential, as well as the *in vitro* stability. Finally, the cytotoxicity and antioxidant capacity of the selected SMEDDS formulation was also examined.

## 2. Results and Discussion 

### 2.1. Solubility of RSV in Various Oils, Surfactants and Cosurfactants

**Figure 1:**
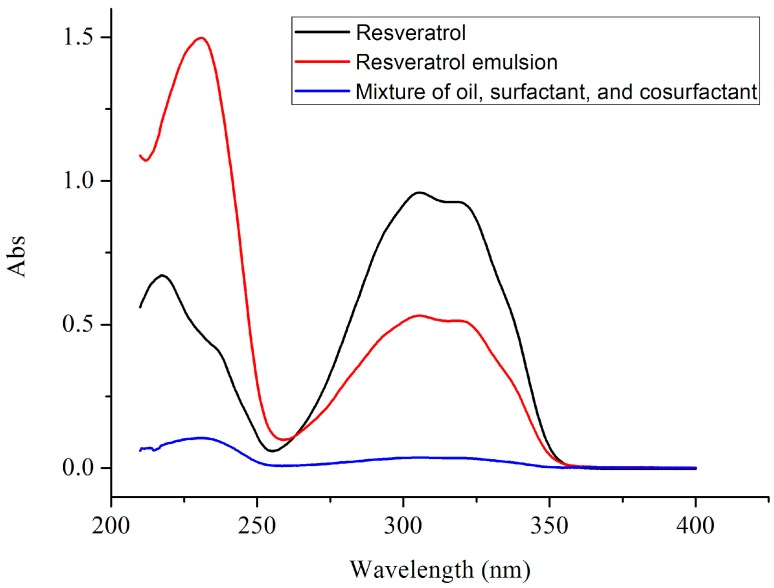
UV spectra of RSV, RSV emulsion, and mixture of oil, surfactant, and cosurfactant.

A suitable oil, surfactant, and co-surfactant are pivotal to the development of poorly water-soluble RSV. In the present study, three oils (ethyl oleate, castor oil, and olive oil), two surfactants (Triton X-100 and Tween-80), and three co-surfactants (glycerol, glycol ether, and PEG-400) were used to measure the solubility values of RSV. Figure 1 shows that RSV exhibited a UV absorption peak at 306 nm, which is the typical absorption maxima usually displayed by flavonoids compared with the oil, surfactant, and co-surfactant mixture [17]. In Table 1, olive oil exhibited a high RSV solubility (7.23 ± 1.54 mg/mL), but it was lower than that in ethyl oleate (60.84 mg/mL). Among the three oils, castor oil exhibited the lowest solubility of RSV (5.95 ± 0.82 mg/mL). In the surfactant screening study, the solubilities of RSV in Triton X-100 and Tween-80 were 23.22 mg/mL and 7.30 ± 2.12 mg/mL, respectively. The solubility of RSV in the tested co-surfactants was as follows in decreasing order: glycol ether > PEG 400 > glycerol (35.17 ± 6.83 mg/mL, 33.22 ± 3.45 mg/mL, 21.08 ± 4.08 mg/mL, respectively). Because the hydrophilic-lipophilic balance (HLB) value of the surfactant determined the efficiency of self-microemulsification, surfactants with HLB 12–15 were used to show good efficiency. Considering that the safety and biocompatibility of Tween-80 and PEG 400 is superior to Triton X-100 and glycol ether, the former has been selected as the surfactant and co-surfactant. As a result, ethyl oleate, Tween-80, and PEG 400 were used as the oil, surfactant and co-surfactant candidates, respectively, for the optimal SMEDDS formulation. RSV was encapsulated at a final concentration of 5% by weight (*w*/*w*) for further studies.

**Table 1:** Solubility of RSV in different vehicles.

Excipient	Solvent	Solubility (mg/mL)
Oil	Ethyl oleate	60.84 ± 5.28
	Castor oil	5.95 ± 0.82
	Olive oil	7.23 ± 1.54
Surfactant	Tween 80	7.30 ± 2.12
	Triton X-100	23.22 ± 4.19
Co-surfactant	PEG 400	33.22 ±3.45
	Glycerol	21.08 ± 4.08
	Glycol ether	35.17 ± 6.83

### 2.2. Phase Diagram Construction

**Figure 2:**
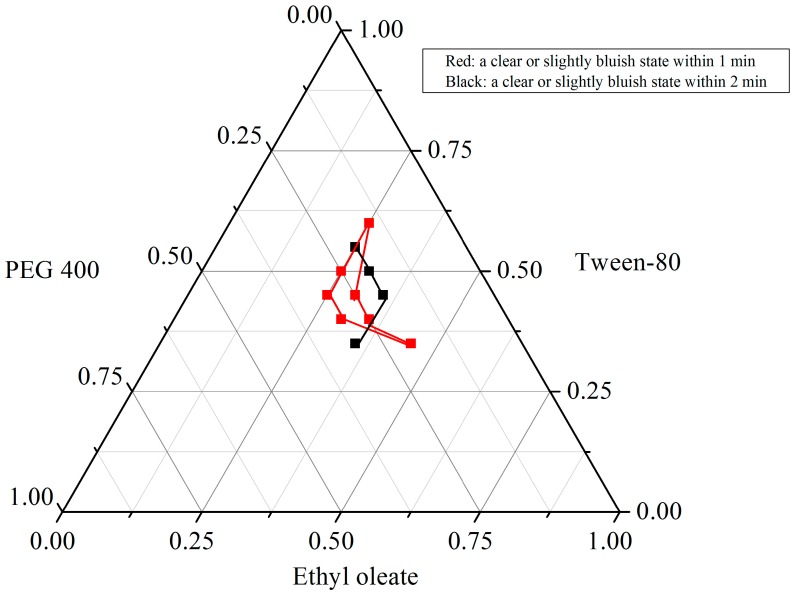
Pserdo-ternary diagram of ethyl oleate (oil), Tween 80 (surfactant), and PEG 400 (co-surfactant).

In the GI tract, the more rapid the self-emulsion, the more clearance and less oily appearance that can be seen. Thus, enhancing the self-emulsion efficiency in aqueous solution is a key consideration in designing these formulations by forming small droplet size and homogeneous states. Commonly, parameters determining the self-emulsifying ability included the emulsification time and solution clearance. From our results (Figure 2), only seven good grade (a clear or slightly bluish state within 1 min) formulations were obtained from the 49 formulations were obtained, which are 25:45:30, 25:50:25, 25:60:15, 30:40:30, 30:45:25, 35:40:25, and 45:35:20 (*w*/*w*/*w* of oil, surfactant and co-surfactant). Notably, among all of the 49 formulations, both when the surfactant varied from 35% to 60% (*w*/*w*) and the co-surfactant varied from 15% to 30% (*w*/*w*), fine micro-emulsions were formed, most of which were given relatively good visual grades (a clear or slightly bluish state within 2 min). However, when the weight ratio of oil exceeds 45% (*w*/*w*), a poor capacity for micro-emulsion formation has been observed (dark gray and slightly oily appearance even a large number of oil droplets more than 3 min). Contrary to the previous research which showed a high concentration of oil is beneficial for improving solubility, in the present study, a larger oil concentration indicated less micro-emulsion in the oil-in-water formation.

### 2.3. Characteristics of RSV Emulsion

**Figure 3:**
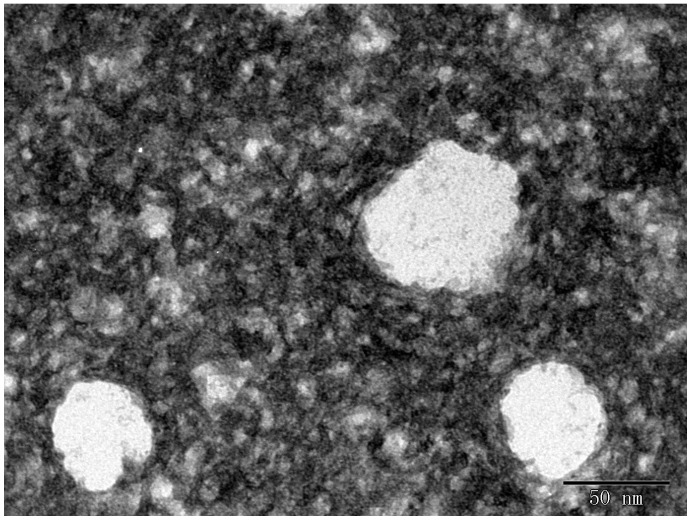
TEM images of encapsulated RSV particles. Particles have similar morphological aspects with spherical shapes and no aggregation.

In previous studies, Sessa *et al.* showed that the diameter of microencapsulated RSV was over 200 nm in oil-in-water formulations [9]. The droplet sizes of the good SMEDDS formulations measured by dynamic light scattering (DLS) are presented in Table 2. Unexpectedly, our results showed that the droplet sizes ranged from 48.66 nm to 58.36 nm, which were smaller than those that have been reported. Moreover, these nanoparticle dispersions exhibited a unimodal distribution, and PDI values narrowly ranged from 0.109 to 0.198, indicating typical monodispersed systems. Meanwhile, the morphology of the self-emulsion formed from this encapsulated formulation was detected by using TEM, which showed spherical shapes and uniform droplet size of the self-emulsion without aggregation (Figure 3). Considering that a higher oil concentration could increase RSV loading and a high concentration of surfactant was required for stable self-emulsion formation in pH 1.2 dissolution conditions, the formulation (35:40:25 *w*/*w*/*w*) was selected for further experiments.

**Table 2:** Droplet size (d) with the corresponding PDI of encapsulated RSV in different ratios of ethyl oleate, Tween 80, and PEG 400.

Ethyl Oleate:Tween 80:PEG 400	d (nm)	PDI
25:45:30	51.84 ± 2.32	0.109 ± 0.022
25:50:25	43.97 ± 1.67	0.198 ± 0.015
25:60:15	51.2 ± 2.25	0.127 ± 0.019
30:45:25	58.36 ± 1.98	0.165 ± 0.013
35:40:25	48.66 ± 2.83	0.185 ± 0.017

### 2.4. In Vitro Stability

It was important to determine the nanoparticles’ stability to find out whether the RSV remained encapsulated in the core of the nanoparticles during storage. Most of all nanoparticles have been found stable at 4 °C for up to 60 days with no significant changes to the particle size, which was vise verse at higher storage temperature. In order to assay the physical stability of micro-encapsulated RSV, the mean droplet size of the best formulation was measured at 30 °C for 30 days. As shown in Table 3, the initial droplet diameter of encapsulated RSV was 48.66 nm. Notably, the diameter had no statistically significant changes with the time for up to 30 days. This long term stability study indicated that this encapsulated RSV is stable and might have good dispersion quality in long term storage.

**Table 3:** Evaluation of the mean droplet size of encapsulated RSV (of ethyl oleate, Tween 80, and PEG 400) at 30 °C for 30 days.

Days	d (nm)	PDI
0	48.66 ± 2.83	0.185 ± 0.017
10	50.2 ± 1.26	0.108 ± 0.021
20	49.32 ± 2.05	0.173 ± 0.012
30	50.08 ± 2.01	0.145 ± 0.016

However, the low stability of this encapsulated RSV in acidic conditions may be a major obstacle in its formulation. In order to explore the alteration of the droplet size in the transition from stomach to intestine following oral administration, we incubated the RSV emulsion in simulating gastric fluid (HCl solution, pH 1.2) and simulated intestinal fluid (PBS, pH 7.4) environment at 37 °C. It seemed that the different pH medium had effect on the droplet size (Table 4) and self-micro-emulsifying behaviour. In line with the research of Bolko *et al.* who demonstrated that the droplet size was approximately 30 nm by using mixed glycerides as the lipid phase, we found that self-emulsion particles exhibited a mean globule size <35 nm with a narrow distribution in *in vitro* neutral and acid environments [13]. However, there is a significant difference with water medium, suggesting that this formulation can form small nanoparticles in physical circumstances (*p* < 0.05). The globules were robust to dilution as they did not show any phase separation and drug precipitation even after 24 h of storage (data not shown).

**Table 4:** Droplet size (d) with the corresponding PDI and zeta potential of the optimal formulation (ethyl oleate, Tween 80, and PEG 400 at a ratio of 35:40:25).

Medium	d (nm)	PDI	Zeta Potential (mV)
Water	43.15 ± 3.67	0.207 ± 0.02	−0.2715 ± 0.02
pH = 1.2 (HCl)	34.61 ± 2.87 *	0.197 ± 0.01	−0.3642 ± 0.03
pH = 7.4 (PBS)	33.5 ± 4.06 *	0.129 ± 0.01	−0.1091 ± 0.03

* *p* < 0.05 *vs.* water.

The stability of nanoparticles also depends in part on the surface zeta (ζ)-potential, a parameter which gives the magnitude of the electrostatic repulsive interactions between particles [18]. A high ζ-potential indicates good dispersion stability as charged surfaces prevent the aggregation and fusion of the nanoparticles by electrostatic repulsion. As shown in Table 4, this nanoformulation presented a higher negative average zeta potential of around −0.2 mV, which indicated that these charged ions on the surface of the nanoparticles contributed to the adsorption and physically stability in different pH medium [19].

### 2.5. Cytotoxicity of Encapsulated RSV

**Figure 4:**
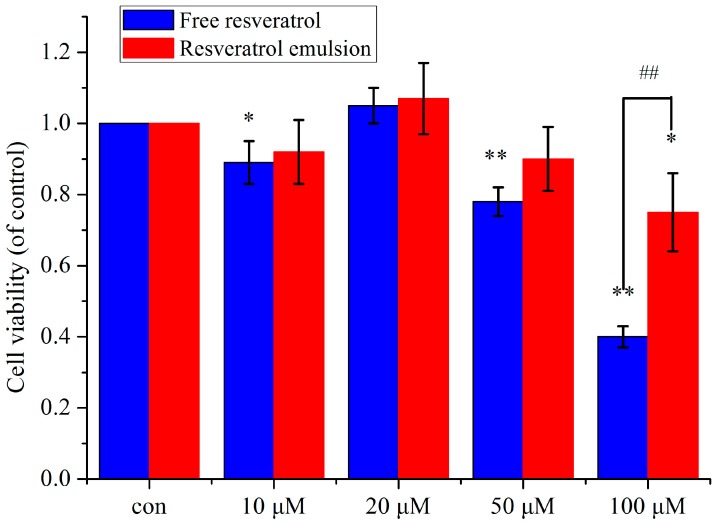
Viability of PC12 cells cultured with free RSV and encapsulated RSV for 48 h at 10, 20, 50, and 100 μM concentrations (*n* = 3). * *p* < 0.05 *vs.* control, ** *p* < 0.01 *vs.* control; ^##^ *p* < 0.01 *vs.* free RSV.

Notably, there are some controversial results regarding the safety of the delivery system [20]. Furthermore, the observed cytotoxicity of RSV itself at a high dose should be taken into account for use as either a nutraceutical or a drug [21,22]. The results revealed less toxicity in RSV nanoparticles (Figure 4). In the encapsulated group, cell viability slightly decreased with increased dosage, among which a significant difference was only observed in the 100 μM concentration (*p* < 0.05). However, free RSV dose-dependently attenuated cell growth, especially at the 100 μM concentration (*p* < 0.01) [23]. Therefore, concentrations below 100 μM were determined as suitable RSV concentrations for encapsulated RSV contrary to the free RSV group (50 μM), suggesting the lower toxicity of encapsulated RSV.

### 2.6. Intracellular Antioxidant Activity

**Figure 5:**
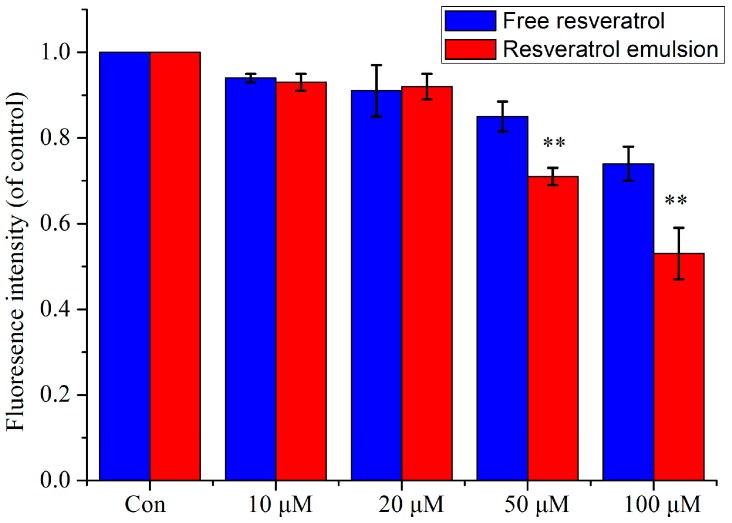
Intracellular antioxidant activity of encapsulated and free RSV at different concentration (*n* = 3). ** *p* < 0.01 *vs.* free RSV.

Under physiological conditions, DCFH-DA can be easily transported trans-membrane into cells forming non-fluorescent DCFH. The intercellular DCFH can be oxidized to fluorescent DCF by ROS, which directly indicated the intracellular ROS level. H_2_O_2_ is a biologically relevant reactive oxygen species and is readily diffused into the cells. Once inside the cells, H_2_O_2_ oxidizes the intracellular DCFH to the fluorescent DCF. In addition, H_2_O_2_ has long been used to induce intracellular ROS generation [24]. However, the nanoencapsulated RSV exerts its cellular antioxidant activity by preventing the oxidation of DCFH and reducing the formation of DCF. Due to the fact that drugs, which existed in the medium, have been removed, under these circumstances, a lower ROS level might indicate that more drugs entered into cells and has a better antioxidant activity. Considering the neuroprotective effects of RSV and RSV nanoparticles being used to improve the neuroprotective effects against Parkinson’s disease, in the present study, the antioxidant capacity of encapsulated and free RSV were measured by using PC12 cells. From Figure 5, a strong dose dependent cellular antioxidant activity of the free RSV groups can be observed. However, in nanoencapsulated group, the antioxidant capacity was significantly increased in a dose dependent manner compared with free RSV group, suggesting that this nanometric size of the particles facilitated the cellular uptake. It has been reported that SMEDDS can form nanosized (<100 nm) emulsion droplets, which increased the intestinal absorption rate and lymphatic uptake of lipophilic molecules. In line with previous researchers who have utilized SMEDDS as a carrier system to enhance the bioavailability of nutraceuticals, in the present study, we also proven that this nanoencapsulated formulation was an effective way to increase cell uptake of RSV, especially at 50 μM and 100 μM concentration (*p* < 0.01 *vs.* free RSV group) [25,26,27].

## 3. Experimental Section

### 3.1. Materials and Chemicals

RSV (>98%) was obtained from the Shaanxi Huike Botanical Development Co., Ltd. (Xi’an, China). Ethyl oleate, castor oil, olive oil, glycerol, glycol ether, Tween-80, Triton X-100, and PEG-400 were purchased from the Sinopharm Chemical Reagent Co., Ltd. (Shanghai, China). Purified water was prepared by a Millipore Milli-Q ultrapure water purification system (Billerica, MA, USA). PC12 cells were obtained from the Cell Bank of the Chinese Academy of Sciences (Shanghai, China). RPMI 1640 medium was purchased from Thermo Fisher Scientific Inc. (Waltham, MA, USA). Horse serum was obtained from HyClone (Logan, UT, USA) and foetal bovine serum was from the Tianhang Biological Technology Stock Co., Ltd. (Hangzhou, China). Penicillin/streptomycin, DCFH-DA, and the CCK-8 kit were purchased from the Beyotime Institute of Biotechnology (Shanghai, China). All of the organic solvents were of analytical grade.

### 3.2. Solubility Study of RSV in Various Oils, Surfactants, and Cosurfactants

First, the solubilities of RSV in various oils, surfactants, and cosurfactants were measured. Briefly, an excess amount of RSV (10 g) was added to a small conical flask containing 20 g of various oils (ethyl oleate, castor oil, and olive oil), surfactants (Tween-80 and TritonX-100), and co-surfactants (PEG-400, glycerol, and glycol ether). After ultrasonication for 30 min, the mixtures were shaken in the dark in an air bath oscillator (THZ-82B, Medical Instrument Factory, Jiangsu, China) for 48 h at 37 °C. After reaching equilibrium, the mixtures were centrifuged at 10,000 rpm for 10 min, and the supernatants were transferred. The concentration of RSV was determined by ultraviolet-visible spectroscopy using a TU-1810PC unit (Purkinjie, Beijing, China) after being filtered through 0.45-μm membrane filters. The RSV concentration was compared with a standard curve of pure RSV.

### 3.3. Ultraviolet-Visible Spectroscopy (UV)

The UV spectra were recorded for RSV using a TU-1810PC UV spectrophotometer (Purkinje). Each sample was dissolved in methyl alcohol at room temperature (25 °C) in the appropriate concentration, so that their UV spectra between the range of 200- and 450-nm could be easily compared.

### 3.4. Phase Diagram Construction

The ternary phase diagrams of mixtures of oil, surfactant, and cosurfactant at certain ratios were constructed. Briefly, according to the solubility results, the optimal oils, surfactants and cosurfactants were mixed in tubes. Then, 1 mL of the mixture was put into 100 mL of water (37 °C) with magnetic stirring to observe the emulsion forming process and final appearance. The boundaries of the self-microemulsification regions in the phase diagrams were determined by connecting the points representing formation of the microemulsion [15,28].

### 3.5. Preparation of the RSV Emulsion Formulation

Based on the micro-emulsion region, the RSV emulsion formulation at the optimal component ratio was prepared. Briefly, 50 mg of RSV was added to the oil with gentle stirring. Then, the desired ratio of surfactant and co-surfactant was mixed. Consequently, RSV containing oil was added into the surfactant and co-surfactant mixture with continuous stirring until homogeneity was achieved.

### 3.6. Droplet Size Determination

RSV emulsion was diluted with distilled water to a suitable concentration and then gently mixed with a magnetic stirrer. After equilibrium, samples were placed in transparent polystyrene cuvettes (1 cm^2^) and loaded in a thermostated sample chamber. The droplet size distribution and polydispersity index (PDI) were detected by using dispersion technology software (Zetasizer 3000HS, Malvern, Worcestershire, UK). The mean particle diameter analysis was carried out by scattering light with a 90° angle at a temperature of 25 °C and was evaluated by using the volume-weighting pattern.

### 3.7. Zeta Potential

The emulsion stability is directly related to the magnitude of the surface charge. The zeta potential of the diluted oil-in-water formulation was measured using a Malvern Zetasizer 3000HS.

### 3.8. Morphology

The morphology of the nanoparticles was observed using a transmission electron microscope (JEM-1200EX, JEOL Ltd., Tokyo, Japan). The SMEDDS formulation was diluted with deionized water at 1:100 and mixed by slight shaking. One drop of diluted sample was deposited on a 300-mesh carbon film-coated grid for 15 min and then stained with a 2% aqueous solution of phosphotungstic acid (PTA) (pH = 7.0). The dried samples were observed under an electron microscope.

### 3.9. Effect of Different Dilution Media on the Resveratrol Emulsion

The influence of different dilution media on the droplet size and zeta potential was studied by diluting the RSV formulation with phosphate buffered saline (PBS, pH 7.4) and HCl (pH 1.2). The self-micro-emulsification efficiency and transparency were visual assessed as stated above. Then, the droplet size and zeta potential were determined as described above.

### 3.10. Cell Culture

PC12 cells were grown at 37 °C in 5% CO_2_ in RPMI 1640 medium supplemented with 10% horse serum, 5% foetal bovine serum, and 1% penicillin/streptomycin. For the RSV treatment groups, the cells were treated with different concentrations of encapsulated RSV.

### 3.11. Cytotoxicity Studies

The cytotoxicity of encapsulated RSV was determined by a CCK-8 kit according to the manufacturer’s instructions. Briefly, 5 × 10^4^ cells suspended in RPMI 1640 medium (100 μL) containing 10% horse serum and 5% FBS were seeded into 96-well plates. Ten microliters of CCK-8 solution was added to each well, and the cells were incubated at 37 °C for 1 h at the end of the experiment. The absorbance was then measured at 450 nm. The background absorbance of the medium in the absence of cells was subtracted. The experiments were independently repeated at least three times.

### 3.12. Measurement of Intracellular Relative Oxygen Species (ROS)

The intracellular ROS levels were measured using the cell permeable non-fluorescent 2′,-7′-dichlorodihydrofluorescein diacetate (DCFH-DA) according to previous reports [29]. 1 × 10^5^ cells were first pre-treated with the nano-encapsulated RSV (10, 20, 50, and 100 μM) for 1 h at 37 °C. Being washed with medium, the cell was incubated with DCFH-DA at a final concentration of 10 μM for 30 min at 37 °C. Then the cells were washed with medium. Finally, H_2_O_2_ was added to final concentration of 50 μM. After being washed with serum-free culture medium for 3 times, the fluorescence was measured with a microplate reader at an excitation wavelength of 488 nm and an emission wavelength of 525 nm every minute for 30 min.

### 3.13. Statistical Analysis

The statistical analysis was performed using the SPSS 17.0 software (Chicago, IL, USA). Data are presented as the mean ± SD derived from three independent experiments. Significant differences were tested with a one-way ANOVA followed by Tukey’s multiple comparison *post-hoc* test. Differences were considered significant at a level of *p* < 0.05.

## 4. Conclusions

This study showed that nanoencapsulated RSV appears to be an interesting approach to solve the problems associated with the poor solubility of RSV. The optimal formulation was ethyl oleate (35%), Tween 80 (40%), and PEG400 (25%) based on the high drug loading and small droplet size parameters. *In vitro* stability studies revealed that the different pH of the media had no effect on ζ-potential, but a small droplet size (approximately 35 nm) was observed in PBS (pH 7.4) which mimics the gastrointestinal environment. More importantly, this stable formulation has greater anti-oxidative capability and less toxicity to cells than free RSV. Taken together, these results along with the simple fabrication process indicate that this formulation can deliver functional food supplements through a stable, efficient, and safe pathway.
